# Brain Functional Reserve in the Context of Neuroplasticity after Stroke

**DOI:** 10.1155/2019/9708905

**Published:** 2019-02-27

**Authors:** Jan Dąbrowski, Anna Czajka, Justyna Zielińska-Turek, Janusz Jaroszyński, Marzena Furtak-Niczyporuk, Aneta Mela, Łukasz A. Poniatowski, Bartłomiej Drop, Małgorzata Dorobek, Maria Barcikowska-Kotowicz, Andrzej Ziemba

**Affiliations:** ^1^Department of Applied Physiology, Mossakowski Medical Research Centre, Polish Academy of Sciences, Pawińskiego 5, 02-106 Warsaw, Poland; ^2^Department Neurology, Central Clinical Hospital of the Ministry of the Interior and Administration, Wołoska 137, 02-507 Warsaw, Poland; ^3^Department of Public Health, 2nd Faculty of Medicine, Medical University of Lublin, Chodźki 1, 20-093 Lublin, Poland; ^4^Department of Experimental and Clinical Pharmacology, Centre for Preclinical Research and Technology (CePT), Medical University of Warsaw, Banacha 1B, 02-097 Warsaw, Poland; ^5^Department of Neurosurgery, Maria Skłodowska-Curie Memorial Cancer Center and Institute of Oncology, W. K. Roentgena 5, 02-781 Warsaw, Poland; ^6^Department of Information Technology and Medical Statistics, Faculty of Health Sciences, Medical University of Lublin, Jaczewskiego 4, 20-090 Lublin, Poland; ^7^Department of Neurodegenerative Disorders, Mossakowski Medical Research Centre, Polish Academy of Sciences, Pawińskiego 5, 02-106 Warsaw, Poland

## Abstract

Stroke is the second cause of death and more importantly first cause of disability in people over 40 years of age. Current therapeutic management of ischemic stroke does not provide fully satisfactory outcomes. Stroke management has significantly changed since the time when there were opened modern stroke units with early motor and speech rehabilitation in hospitals. In recent decades, researchers searched for biomarkers of ischemic stroke and neuroplasticity in order to determine effective diagnostics, prognostic assessment, and therapy. Complex background of events following ischemic episode hinders successful design of effective therapeutic strategies. So far, studies have proven that regeneration after stroke and recovery of lost functions may be assigned to neuronal plasticity understood as ability of brain to reorganize and rebuild as an effect of changed environmental conditions. As many neuronal processes influencing neuroplasticity depend on expression of particular genes and genetic diversity possibly influencing its effectiveness, knowledge on their mechanisms is necessary to understand this process. Epigenetic mechanisms occurring after stroke was briefly discussed in this paper including several mechanisms such as synaptic plasticity; neuro-, glio-, and angiogenesis processes; and growth of axon.

## 1. Introduction

According to the new definition of stroke by the AHA/ASA from 2013, it includes any objective evidence of permanent brain, spinal cord, or retinal cell death due to a vascular cause [1]. In clinical terms, stroke is diagnosed when neurologic deficit in a form of speech, visual disturbance, muscle weakness, or cerebellar dysfunction lasts more than 24 h. In case of symptoms lasting for a shorter period of time, transient ischemic attack (TIA) is diagnosed provided without focus of ischemia in neuroimaging exams [2]. Terms utilizing duration of neurologic symptoms are currently being redefined with use of high-tech imaging methods such as magnetic resonance imaging (MRI) with implementation of diffusion-weighted imaging (DWI) where early ischemic lesions demonstrate increased water level in echo-planar imaging [3]. Pathophysiology definition of ischemic stroke occurs when the blood flow to an area of the brain is interrupted, resulting in some degree of permanent neurological damage [4]. The common pathway of ischemic stroke is lack of sufficient blood flow to perfuse cerebral tissue, due to narrowed or blocked arteries leading to or within the brain. Ischemic strokes can be subdivided into thrombotic and embolic strokes [5]. It is estimated that stroke is the second cause of death after cardiovascular disease and cancer in both low - and high-income countries [6]. Furthermore, ischemic strokes constitute approximately 80% of all strokes [7]. Ischemic strokes can be subdivided into thrombotic and embolic strokes [8]. It is emphasized that pharmacological actions aiming at limiting the area of damage should also include maintaining protective functions of neurons and endothelial cells of vessels composing neurovascular units [9]. Stroke management changed significantly what constitutes natural course of modern stroke units, better medical care, and more targeted motor and speech rehabilitation engaged in the early stage [10]. Increasingly fibrinolytic treatment with recombinant tissue plasminogen activator (rt-PA) and embolectomy are used [11, 12]. There is no commonly accepted therapy targeted on neuroplasticity [13]. During the last decades, researchers searched for indicators of ischemic stroke and neuroplasticity in order to determine effective diagnostics, prognostic assessment, and therapy [14, 15]. Interest of biomarkers has begun since introduction of thrombolytic treatment possible to administer up to 4.5 h from onset of symptoms and in individual cases up to 6 h after fulfilling inclusion and exclusion criteria towards standards of management in acute ischemic phase—according to the American Heart Association (AHA)/American Stroke Association (ASA) [16].

### 1.1. Neuroplasticity

The brain is a complex network of various subsets of cells that have the ability to be reprogrammed and also structurally rebuild [17]. The main point of neuroplasticity is capability of stimulation by a variety of stimuli for modulation of brain activity [18]. Brain compensates damages through reorganization and creation of new connections among undamaged neurons [19]. After ischemia of cells, oxygen deprivation in neurons cascades destruction in focus of infarction being formed lasts for many hours, usually leading to progression of damage [20].

### 1.2. Future Approach

Future research will be focused on markers of brain damage and could aid in understanding mechanisms disturbing plasticity. One of these may be inflammatory reaction initiated immediately after stroke leading to neuron damage but also possibly demonstrating neuroprotective activity [21]. The scientists from the University of California, Harvard University, and Federal Polytechnic in Zurich provided that after injury of the spinal cord exists the increased expression on genes leading to growth of damaged axons in mice and rats [22].

### 1.3. Focus of Ischemia: Pathology

Ischemic stroke occurs as a result of two primary pathological processes including oxygen loss and interruption in glucose supply to specific brain regions [23]. Inhibition of energy supplies leads to dysfunction of neurotransmission [24]. It was observed that disturbance of neuron functions occurs when cerebral flow decreases to 50 ml/100 g/min [25]. Irreversible damage occurs when cerebral blood flow decreases consecutively to 30 ml/100 g/min [26]. The level and duration of decreased flow are associated with increasing probability of irreversible neuron damage [27]. In an event of blood flow arrest in cerebral tissue, neuronal metabolism is disturbed after 30 seconds, whereas in consecutive minutes of oxygen deficiency, cascade reaction begins, eventually leading to brain infarction [28, 29]. Among occurring reactions included are as follows: local dilation of vessels, circulatory disturbances in vessels, local swelling, and necrosis [30]. Alternations on the neuronal level lead to disturbed functional activity of cells and their apoptosis [31]. These disturbances originate from dysfunction of Na^+^/K^+^ATPase leading to depolarization of neuronal membrane, releasing excitatory neurotransmitters and opening of calcium (Ca^2+^) channels [32]. Secondary damage of neurons and cell organelles and further dysregulation of cellular metabolism occur [33]. In this case, Ca^2+^ ions spread intracellularly through channels gated with potential or receptors that may be additionally induced by several neurotransmitters in excitotoxicity mechanism [34, 35]. More delayed processes accompanying stroke are related to the neuroinflammatory process and cellular apoptosis initiated within a number of minutes after ischemic attack and may last for even several weeks and months [36]. These events may lead to delayed death of neurons and are subject of vast research concerning neuroprotective theories and agents [37]. Complex background of events following an ischemic episode hinders successful design of effective therapeutic strategies. Current research is directed at neuroprotective and proregenerative therapies which may aid recovery of lost functions by neurons after an ischemic episode. Studies have proven that regeneration after stroke and recovery of lost functions may be assigned to neuronal plasticity understood as the ability of the brain to reorganize and rebuild as an effect of changed environmental conditions [38]. It is well known that ischemic stroke triggers inflammatory cascade through activation of numerous cell mediators. Ischemia leads to accumulation of glutamate (Glu) in extracellular space and excitotoxicity [39]. In ischemic tissue, reactive oxygen species are generated and blood-brain barrier (BBB) integration is significantly disturbed [40]. Microglia are the first line of cells reacting on damage and primary source of proinflammatory cytokines and chemokines [41, 42]. Their release causes local activation of microglia, intensification of cell adhesion, and mobilization of leukocytes [43]. Increased oxidative stress and cytokine activation contribute to further intensification of inflammatory process including regulation of matrix metalloproteinase (MMP) from astrocytes and microglia leading to BBB dysfunction and eventually death of neurons [44]. Ageing decreases capabilities of neurons for functional plasticity in a healthy brain [45]. Regaining lost functions may be explained by neuronal plasticity and decreased ability for reorganization possibly being a significant factor causing a worse functional result in elderly patients [46]. For better understanding of neuroplasticity, tracking of genetic changes influencing it is needed [47]. As many neuronal processes influencing neuroplasticity depend on expression of particular genes and genetic diversity possibly influencing its effectiveness, knowledge on their mechanisms is necessary to understand this process. Epigenetic mechanisms occurring after stroke will be briefly discussed in this paper. Background of epigenetic changes is characterized by several mechanisms such as synaptic plasticity; neuro-, glio-, and angiogenesis processes; and growth of axon. Each of these processes is modified molecularly through DNA methylation, histone modification, and microRNA (miRNA) actions.

## 2. Neuronal Plasticity

Synaptic plasticity is achieved through improvement of communication in synaptic connections between existing neurons and is fundamental for retaining neuronal networks [48]. Its very important information surrounding the focus of ischemia is the existing area name penumbra. Immediately following the event, blood flow and therefore oxygen transport are reduced locally, leading to hypoxia of the cells near the location of the original insult. This can lead to hypoxic cell death (infarction) and amplify the original damage from the ischemia; however, the penumbra area may remain viable for several hours after an ischemic event due to the collateral arteries that supply the penumbral zone. As time elapses after the onset of stroke, the extent of the penumbra tends to decrease; therefore, in the emergency department, a major concern is to protect the penumbra by increasing oxygen transport and delivery to cells in the danger zone, thereby limiting cell death. The existence of a penumbra implies that salvage of the cells is possible. There is a high correlation between the extent of spontaneous neurological recovery and the volume of penumbra that escapes infarction; therefore, saving the penumbra should improve the clinical outcome [49]. Epigenetic regulation, which involves DNA methylation and histone modifications, plays a critical role in retaining long-term changes in postmitotic cells. Accumulating evidence suggests that the epigenetic machinery might regulate the formation and stabilization of long-term memory in two ways: a “gating” role of the chromatin state to regulate activity-triggered gene expression and a “stabilizing” role of the chromatin state to maintain molecular and cellular changes induced by the memory-related event. The neuronal activation regulates the dynamics of the chromatin status under precise timing, with subsequent alterations in the gene expression profile.

### 2.1. DNA Methylation

In the study of Levenson and Sweatt in 2005, they proved that DNA methyltransferase enzyme family (DNMT) is important for synaptic plasticity [50]. Inhibition of DNMT activity causes long-term blockade of hippocampus potentiation and leads to decreased methylation of protein promoters called reelin, brain-derived neurotrophic factor (BDNF), and other genes participating in synaptic plasticity. Increased excitability within the penumbra is associated with dynamic regulation of DNA methylation [51, 52]. One of the most interesting phenomena is the process of active demethylation of gene promoter regions of BDNF through growth arrest and DNA-damage-inducible beta (GADD45B) protein activity. The role of GADD45B as a key DNA demethylation coordinator is mostly based only on in vivo experiments; however, it is difficult to distinguish active from passive demethylation of DNA. N-Methyl-D-aspartate (NMDA) agonism is found to induce expression of GADD45B mRNA and BDNF, at the same time reducing mRNA expression [53]. Ma et al. in 2009 documented that BDNF IXa is demethylated by GADD45b in mice [54]. Although there are regulatory differences between human BDNF IXabcd and mouse BDNF IXa [55], there also exist several similarities. In vivo and in vitro human BDNF IXabcd and mouse BDNF IXa are similarly induced by neuronal activity [56].

### 2.2. Histone Modifications

Histone modifications protruding from the nucleosome core are acetylated or deacetylated. It is an epigenetic mechanism for controlling gene expression. A very important epigenetic mechanism for controlling gene expression is posttranslational modification of histones. In that modification, the rest of lysine at the N-terminus are acetylated or deacetylated. The function of histone lysine deacetylase (HDAC2) is not limited to long-term synaptic potentiation; it also includes creation of memory in the hippocampus [57]. In anatomical terms, inhibition of HDAC2 significantly increases creation of dendritic bridges in neurons of the hippocampus. It is now evident that integration and regulation of epigenetic modifications allow for complex control of gene expression necessary for long-term memory formation and maintenance. Dynamic changes in DNA methylation and chromatin structure are the result of well-established intracellular signaling cascades that converge on the nucleus to adjust the precise equilibrium of gene repression and activation [58].

### 2.3. miRNA

miRNAs are endogenous, noncoding RNAs that take part in the posttranscriptional regulation of gene expression mainly by binding to the 3-untranslated region of messenger RNAs (mRNAs). A few of miRNAs which are isolated from brain play an important role in synaptic plasticity. They also take important part in learning and memory function [59].

Activity-regulated cytoskeleton-associated protein (ARC) gene is an important regulator of synaptic plasticity. Its expression is decreased in the ischemic cortex and significantly increased in the tissue cortex surrounding ischemic focus shortly after stroke, probably as an effect of Glu release and activation of neurons [60]. ARC expression is regulated by multiple miRNAs and ectopic miRNA expression in hippocampal neurons and by inhibition of the endogenous miRNAs in neurons. Frisén in 2016 proved that during in vitro neuronal development, there is an inverse relationship between ARC mRNA expression and expression of ARC-targeting miRNAs. Thus, at DIV10, expressions of miR-19a, miR-34a, miR-326, and miR-193a were decreased while ARC mRNA was elevated [61].

## 3. Neuro-, Glio-, and Angiogenesis

Taking into consideration that synaptic plasticity is achieved through improvement of communication in synapses between existing neurons, the terms neuro-, glio-, and angiogenesis refer to development and formation of new neurons and blood vessels in the brain [62–64]. Recently, it has been proved that formation of new neurons is not limited to the time before birth [65, 66]. However, in order for neurogenesis to occur, one condition must be fulfilled, that is, presence of stem cells and progenitor cell and special types of cells in the dentate gyrus, in the hippocampus, and possibly in the prefrontal cortex which will become completely equipped neuron with axons and dendrites [67, 68]. New neurons can migrate to distant areas of the brain to fulfill important and previously lost functions [69]. Neuronal death is a strong stimulant for neurogenesis after ischemic stroke [70, 71]. Ischemic event is followed by increased formation of cells from these regions and alteration of migration pathways toward damaged area [72–74]. The majority of cells die and very few participate in this process [75]. Recently, researchers use the denomination of “neurovascular unit.” The neurovascular unit involves connection of neurons with blood vessels and involves growth factors influencing neurogenesis which indirectly affect angiogenesis [76]. Neurogenesis and angiogenesis occur after ischemic stroke. It is modulated by DNA methylation, histone modification, and miRNA actions. The formation of long-term memory involves a series of molecular and cellular changes, including gene transcription, protein synthesis, and synaptic plasticity dynamics [77].

### 3.1. DNA Methylation

Methylation silences gene expression in a variety of ways, one of which is recruitment of specific binding proteins to an element of promoter [78]. The family of methyl-CpG-binding domain (MBD) binding proteins include MBD1-4 and methyl-CpG-binding protein 2 (MECP2). The scientists observed increase of MBD1 and MECP2 after 24 hours of stroke, and expression of MBD2 increases after 6 h from ischemia [79]. All mentioned proteins have regulatory functions in neurogenesis process [80]. DNA methylation was recognized in the past as a highly stable gene silencing method. At present, evidence suggests that methylation states may be more dynamic than it was previously assumed [81]. Growth arrest and DNA damage 45 (GADD45) proteins are significant elements of active cytosine (Cys) residue demethylation process [82]. The process is mediated by DNA repair pathway. GADD45 may function through feedback of necessary enzymatic process in which demethylation could lead to increased expression of specific genes significant for neuroplasticity.

### 3.2. Histone Modifications

Particular elements of polycomb-group proteins participate in neurogenesis [83]. Formation of oligodendrocytes is also transformed during stroke with histone deacetylase. It is already in the acute phase of stroke that oligodendrocyte progenitor cells (OPC) in the white matter of penumbra demonstrate increased protein expression of HDAC1 and HDAC2 along with increased proliferation [84]. What is more particular, HDAC isoforms may have diverse impact on cell maturation [85]. In their study, Wang et al. in 2012 demonstrated that valproic acid (VPA), a strong histone deacetylase inhibitor, has impact on regaining functions after stroke. That acid additionally increases the density of blood vessels thus improving cerebral blood flow to the ischemic hemisphere 14 days after stroke [86]. It was also demonstrated that VPA mediates in regeneration through promoting neuronal diversity in hippocampus progenitor cells [87].

### 3.3. miRNA

The role of miRNA was widely recognized as a regulator in neurogenesis. As previously stated, miR-124 is important in the acute phase of stroke. This is a ligand Jagged1 (JAG1) targeting as a neuronal determinant in the normal subventricular zone (SVZ) [88]. That miRNA influences repair after stroke through regulation of behavior of progenitor cells. In the brain with ischemia, miR-124 is reduced in the SVZ for 7 days after stroke that corresponds with time of significant neurogenesis [89]. Another miRNA transcript potentially important for brain repair after stroke is miR-9 [90]. Its loss inhibits proliferation in human neuronal progenitor cells and intensifies migration of these cells after transplantation to the ischemic brain [91].

## 4. Axon Growth

The growth of the axon becomes the main requirement for plasticity and recovery of lost functions. The axon regrowth depends on several neurobiological modifications such as the level of myelination and synapse formation. Despite the ability of axons to grow by altering the extracellular and intracellular substances, dedifferentiation in which axons are responsible for recovering functions from those that are functionally silent is still a matter of discussion. In this case, the intuitive translation relation between anatomical and functional regeneration is questioned [92]. In ischemic stroke as well as in brain injury, the area of brain damage is characterized by the formation of glial scar, in which growth inhibitors are upregulated; preventing the effective regeneration of this scar is characterized by significant upregulation of proteoglycans, preventing the effective regeneration of axons. In the close proximity to the glial scar, however, there is a cortical area that is characterized by the expression of many growth-promoting factors that allow axonal growth [93]. One of the main components of glial scars is extracellular matrix proteins known as CSPG, which consist of protein chains and glycosaminoglycans (GAGs). CSPGs are present in the developing and also adult central nervous system, but their expression significantly increases after injury. Reactive astrocytes are responsible for the production and secretion of many CSPGs after injury, and their increased expression is observed for many months. Two reasons for the failure of the CNS regeneration are extrinsic inhibitory molecules and poor internal growth ability [94].

### 4.1. DNA Methylation

Descriptions of the mechanism of DNA methylation in axon growth regulation after stroke are based on published postinjury models; we do not have any models of ischemia [95]. Therefore, recreation of ischemic conditions is difficult. An important role in promoting axon number growth has been recently attributed to proline-rich protein (SPRR1) released after axotomy [96]. High concentration of SPRR1 is released in the cortex of ischemic focus in the initial phase of stroke.

### 4.2. Histone Modifications

SPRR1 may be induced by hypomethylating agents and its expression may be modulated by histone modification [97]. Similarly to the impact of 5-azacytidine on keratinocytes, SPRR1 expression is increased in these cells after treatment with an HDAC inhibitor such as sodium butyrate. Nowadays, we do not have examinations on the human brain [98]. Growth-Associated Protein 43 (GAP43) consists of protein related with a growth cone promoting growth of axons through regulation of cytoskeleton organization with protein kinase C signaling. That expression is strongly induced in the ischemic cortex after ischemic stroke [99]. According to Yuan et al. in 2001, administration of VPA may induce expression of GAP43 as well as of other growth proteins simultaneously promoting regeneration of axons [100].

### 4.3. miRNA

The most important role in the growth of axon is played by miRNA [101, 102]. The role of miR-9, whose level is reduced in the ischemic white matter, is best known. Therefore, miR-9 is released in primary axons of the neuron cortex of a developing brain. miR-9 replicates microtubule-associated protein 1B (MAP1B) connected with a cytoskeleton [103]. That inhibition occurs through RNA interference resulting not only in a significantly increased length of axon but also in a decreased pattern of branches. As in the case of two previous processes, the issue of growth of axon requires further research.

## 5. Discussion

Neuroplasticity is a widespread phenomenon in the function of the nervous system. Spontaneous recovery is the norm in the early poststroke period. Cortical reorganization is common and necessary for postbrain injury recovery. Representations of sensory and motor cortical areas may be modified by the inflow of environmental stimulation during learning and memory processes. Physiotherapy strategies used during recovery process affect the spontaneous neuroplasticity. After stroke, the main functional dysfunctions are aphasia and hemiplegia. Regarding the dynamic changes of a clinical picture of a patient after an ischemic episode, multiplicity, and diversity of pathology, the doctors, physiotherapists, and speech therapists do not have a universal procedure or concept.

A correct therapy depends on the actual deficit and patient necessity [104, 105]. Neural plasticity allows progress of the central nervous system under the influence of variable conditioning environment, learning, and memorization; the new abilities and adaptation into changes happen inside and outside of entourage and activity compensatory process after ischemia. It happens because of a neuron's property enabling overlap indicating changes in the neuronal system in response to organism's needs and challenge of reality [106]. Daily activity, learning, and training have a main influence on brain function. Developing right connections through axons, projections, synapse, and chemical transmitter is an ongoing intricate process with different intensities all throughout the human life. His course determines the information written in the DNA. Genetic predisposition is modified as a result of experience; throughout human life, through environmental changes, the number of synaptic pathways can rise. Many new emerging neuronal cells succumb apoptosis—programmed and irreversible autodestruction—and pruning. The elements of neurons, for example, mitochondria in apoptotic bodies, are removed by macrophages or absorbed through familiar cells. Overproduction of neurons is necessary to obtain an appropriate number of synaptic pathways that kill these cells who cannot create connection functional active [107].

A properly carried out treatment achieves skills by allowing rehabilitation to move beyond the walls of the hospital or home, and this contributes to the functional independence of patients.

Researchers and therapists are still looking for a new possibility of impact of the neuronal system; it will contribute in the future to the functional progress and usage capacity of the mechanisms of neuronal plasticity in the case of his damage [108].

In many sciences, it was confirmed a fact that in regular methodical learning, we can considerably increase intellectual capacity and correct memory, concentration, and logical thinking, and in the case of neurological disorder by targeting the process of neural compensatory plasticity, we can obtain significant improvement of disturbed performance. The effects of neural plasticity depends on the clinical factor, age, intellect, and education of patient. In well-educated people, there exists cognitive reserve of the brain, which may have an impact on the recovery process [109].

A small group of scientists studied Albert Einstein's brain in search of special abilities in the structure of the neuronal system. They compared with another four brains from other people who died at the same age. They discovered in the brain of Albert Einstein a difference in the cytoarchitecture when compared with brains of other people. They found out a higher ratio of astrocytes to neurons in the cerebral cortex parietal lobe in the left hemispheres. Glial cells enable provision of nutritional substances to the brain through connection with the blood vessel, from which we conclude that astroglia can be a ground for neural plasticity [110]. Among many methods of streamlining patients with hemiplegia, we use proprioceptive neuromuscular facilitation (PNF), neurodevelopmental treatment (NDT)/Bobath, constraint-induced movement therapy (CIMT), training oriented on top of approach task-oriented training, neuromuscular arthroskeletal plasticity (NAP), and occupational therapy based on the aim with the rule SMART—specified, measured, attractive, real, and timely. We must select every time a therapy which adapts into individual needs and ability of patient [111]. Neural plasticity allows progress of the central nervous system under the influence of variable conditioning environment, learning, and memorization and the new abilities and adaptation into changes happen inside and outside of entourage and activity compensatory process after ischemia. It happens because of the neuron's property enabling overlap indicating changes in the neuronal system in response to the organism's needs and challenge of reality [112, 113].

According to the above considerations and analyses, it should be indicated that an issue of ischemic stroke not only constitutes individual physical and social impairments but also represents significant financial burden for the global health care systems concerning professionally active people in productive age [114–116]. Patients after an ischemic episode frequently become dependent on institutional organization [117]. Return to daily living and professional activity is hindered, often impossible, for these patients, leading to dependence on the closest relatives [118, 119]. Necessity to help a disabled person causes dysregulation of social and professional life of careers [120]. Optimally, clinical experience should be combined with search for new forms of brain functional reserve [121]. Recovery after stroke is a complex phenomenon. In a study of anti-inflammatory strategies that have been effective for recovery in experimental stroke, Liguz-Lecznar and Kossut described that the most important aspect of therapies targeting the immune system will be regulating the balance between the neurotoxic and neuroprotective effects of inflammatory state components [122]. Clinical experience, awareness of the scale of the problem, and molecular research may be used in combination with each other. It may be assumed that combination of new therapies with neurologic rehabilitation could be a new trend in the treatment of patients after stroke. Another stroke-related issue concerns the substantial prevention. The stroke prevention should consist of complex medical and political issues [123]. We strongly believe that issues discussed in this study should allow better understanding of physiological background and other social aspects of escalating problem of stroke indicating future research directions.
